# Digitally supported physical activity counselling for people with chronic back pain: a randomised controlled parallel feasibility study

**DOI:** 10.1186/s12875-025-02742-z

**Published:** 2025-02-27

**Authors:** Nicole Lindner, Nele Kornder, Julia Heisig, Annette Becker, Veronika van der Wardt, Annika Viniol

**Affiliations:** Department of Primary Care, University of Marburg, Marburg, Germany

**Keywords:** Primary health care, Exercise, Treatment adherence and compliance, Patient-centred care, Pain management

## Abstract

**Background:**

Guiding individuals with chronic back pain (CBP) to initiate and adhere to physical activity (PA) remains challenging. The study rationale is based on the need for innovative strategies, like digital tools, to better promote PA. The aim of this study was to evaluate the feasibility and acceptability of using the digital consultation app ExPa (Exercise against Pain) to support PA consultations for CBP and its potential for a future effectiveness trial. The ExPa app shows the effect of PA on pain and provides individually tailored support to increase PA.

**Methods:**

In a 2-arm randomised controlled feasibility study, we recruited 9 physicians and 37 CBP patients in Hesse (Germany). Using computer assisted cluster randomisation, 14 patients received ExPa counselling from their physician, while 17 patients received standard treatment. Main outcomes focused on study procedures and software use, with secondary outcome including pre- and post-intervention measurements of PA (International Physical Activity Questionnaire (IPAQ), pain and mood (Short Form-12 (SF-12), Von Korff pain intensity and disability score and Hospital Anxiety and Depression Scale (HADS)). Additionally, project-tailored questionnaires and qualitative interviews assessed study procedures and software performance.

**Results:**

Study procedures were generally feasible. However, they took more time and dropouts as well as missing data presented challenges. This provided valuable insights for planning an effectiveness trial. Quantitative and qualitative data indicated that ExPa could have benefits for increasing PA and reducing pain.

**Conclusions:**

Results from the feasibility study indicate that improved procedures are necessary for a larger RCT. ExPa shows potential for positively impacting pain and PA.

**Supplementary Information:**

The online version contains supplementary material available at 10.1186/s12875-025-02742-z.

## Background

Back pain is an extremely common problem, affecting a majority of individuals at some stage in their lives. The global prevalence and economic burden of chronic back pain (CBP) are enormous: Low back pain is the leading cause of activity limitation and work absence among people of all ages and socioeconomic strata [1]. More than a third of community-dwelling adults in the US experienced back pain within a period of three months, with patients facing back pain contributing a staggering $365 billion to overall medical costs in the country [2]. Almost one in three adults in Germany suffers from back pain frequently or constantly [3].

There is extensive evidence that different types of physical activity (PA) lead to improvement in CBP (a.o. pain severity, physical function, psychological function and quality of life) [4]. Numerous clinical guidelines recommend PA as the primary treatment for CBP [5, 6, 7, 4, 8]. Research has demonstrated that the quality of life of individuals with back pain is higher when they engage in higher levels of PA [9].

As we have previously shown, current approaches to promoting PA in primary care are ineffective: our meta-analysis revealed no significant effect of behaviour change interventions to promote PA in primary care [10]. Furthermore, we have shown in a qualitative interview study with physicians and patients with CBP that PA is associated with conflicts for both patients and physicians. Patients with CBP face barriers to engaging in PA, including fear of aggravating their pain and limited access to appropriate exercise resources. Physicians, on the other hand, encounter challenges in providing tailored guidance given the varying levels of patient motivation and concern. This dynamics create a complex doctor-patient relationship, where PA recommendations can sometimes complicate interactions rather than foster a collaborative approach [11]. Physicians face the challenge of counselling and motivating people with CBP to engage in PA. At the same time, people with CBP sometimes have difficulty fully recognising the positive effect of PA on pain and have to manage the integration of PA into their everyday lives without adequate guidance. To address these challenges, mechanisms are needed that enhance physician-patient-communication, support patients in recognising the benefits of PA, and help them integrate PA in their lives. In recent years, there has been a growing recognition of the potential of digital health technologies to address various healthcare needs, including chronic pain management [12]. A systematic review by Valentijn et al. showed that digital health interventions can effectively reduce pain, improve physical functioning and self-management in individuals with musculoskeletal pain. These interventions encompass a broad range of approaches, ranging from patient-focused tools (e.g., personal health tracking and targeted client communication) to professional-focused solutions (e.g., decision support systems, telemedicine, and provider training) and organizational strategies (e.g., health financing and data collection. However, the incorporation of health care providers in the intervention planning remains uncommon. The authors emphasise the need for primary research to explore digital intervention that health care providers can effectively implement [12].

To address this need, we have developed ExPa, a digital communication support programme to strengthen physicians in promoting PA in people with CBP thereby empowering patients to see the benefits of PA and to integrate PA into their lives.

The aim of this study was to evaluate the feasibility and acceptability of using the digital consultation app ExPa (Exercise against Pain) to support PA consultations for CBP and its potential for a future effectiveness trial.

## Methods

### Design

We conducted a cluster randomised controlled mixed method study to assess the feasibility of a study investigating the use of the communication support programme ExPa. In accordance with the guideline on the development of complex interventions this is a phase II study [13]. Design and reporting were guided by the Consolidated Standards of Reporting Trials (CONSORT) extension for randomised pilot and feasibility trials and by TIDieR (Template for Intervention Description and Replication) [14, 15].

The Ethic Board of the University of Marburg approved the study (ethics approval ID: 22/22). We obtained informed written consent from all participants. The protocol followed the tenets of the Declaration of Helsinki [16].

### Setting and participants

We included primary care physicians practising in Hesse (Germany) who worked with a computer in their consulting room and patients with CBP. We applied no additional exclusion criteria. The decision to include only physicians was based on the typical structure of healthcare in Germany, where primary care physicians are usually the point of contact for patients with CBP and are primarily responsible for providing guidance on PA. CBP was defined as back pain on at least 50% of the days for at least three months. We excluded pregnant women, people with cognitive impairment and those who did not speak sufficient German to understand the study, consent to or participate in the study. We applied no additional exclusion criteria. We recruited physicians by telephone or personal contact via the local research practice network. Included physicians identified and recruited patients in their practice. A computer-based cluster randomisation was carried out by a researcher not involved in this project (allocation ratio 1:1). Members of the study team informed participants about their treatment allocation and about the course of the study. Due to the nature of the intervention, blinding of participants and researchers was not possible. Because of the small sample size we did not involve an independent medical statistician.

### Data collection process

Data collection consisted of questionnaires and interviews. Participants received and returned questionnaires by post. Additionally, NL and NK conducted interviews with all physicians and patients of the intervention group either by telephone or in person within a period of three months after the consultation with ExPa.

### Intervention: the digital communication support programme ExPa

We adhered to the UK Medical Research Council (MRC) framework for the development of our complex intervention [13]. The MRC framework emphasises different phases for developing complex interventions. For the first phase (“develop intervention”), we conducted an extensive literature review: Systematic reviews on PA promotion and adherence strategies guided the incorporation of relevant features into ExPa [10]. Additionally, we conducted interviews with physicians and individuals with CBP to assess potential barriers and beneficial factors [11]. We based ExPa on the Behaviour Change Wheel, a well-known framework encompassing various dimensions for long-term behaviour change [17]. For instance, ExPa supports the dimension “education” by providing patients with evidence-based knowledge and the dimension “opportunity” through individually tailored prescriptions for PA. As key stakeholders, a patient advisory board was involved right from the start of the project. Issues raised by members (such as visually demonstrating strength of pain) of the advisory board were factored into the development of the intervention.

**Fig. 1:**
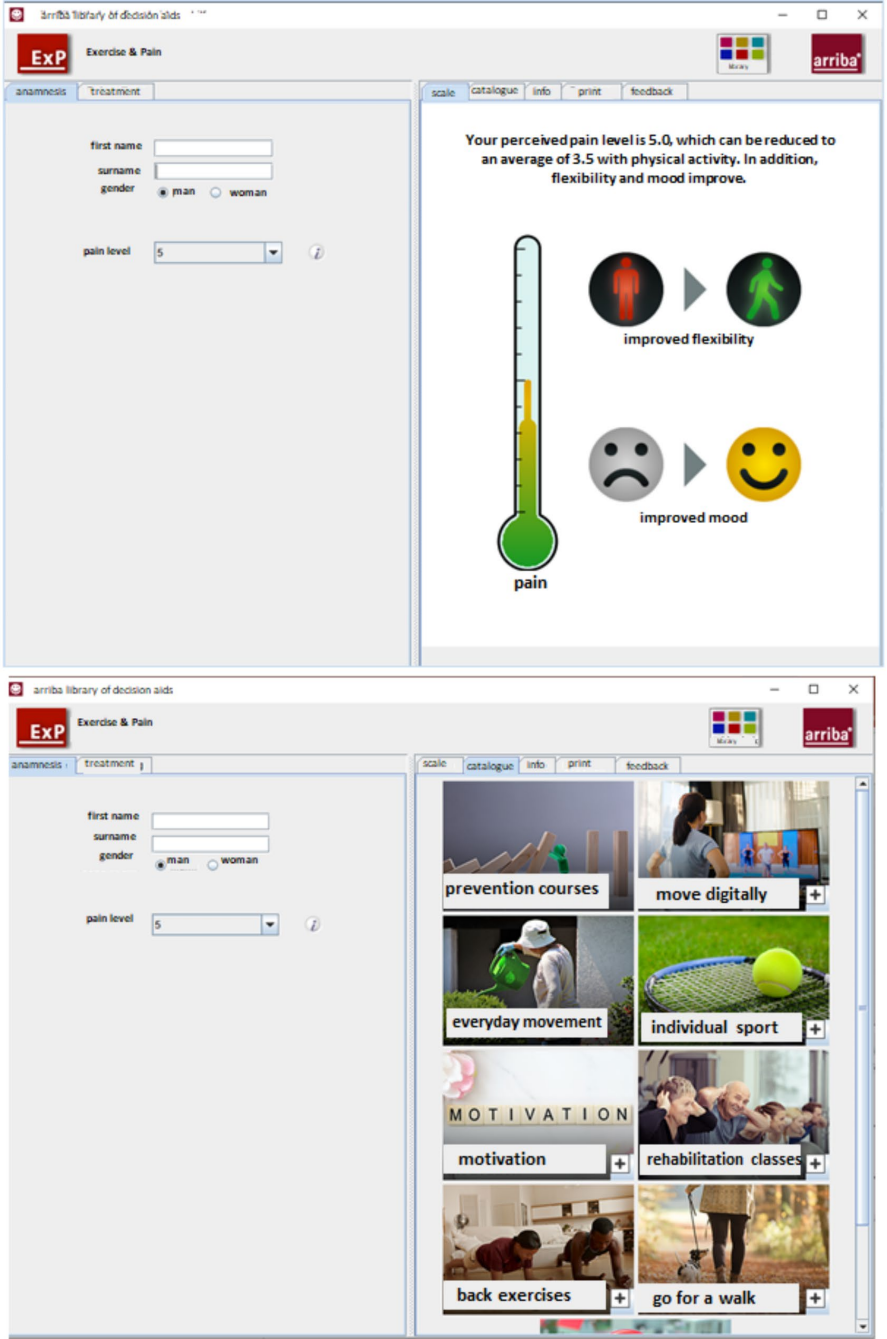
Impressions of the digital counselling software ExPa. ExPa supports physicians in promoting PA in people with CBP by addressing multiple aspects of patient counselling (a.o. enhance physician-patient-communication, support patients in recognising the benefits of PA, and help them integrate PA in their lives). At the top is the screen showing the positive effects of PA on pain, mood and flexibility. At the bottom is the screen showing a catalogue of options to increase PA. The whole software is German as we conducted the study in German primary care practices. For publication, we translated the software in English

The ExPa intervention is based on a software designed for use during consultations to illustrate the positive effect of PA on pain and to suggest personalised strategies to increase PA. In addition, it includes training materials on patient-centred care for physicians, providing ongoing support and resources. In a 15-minute consultation during the regular consultation hours, the physician first assesses pain, visually demonstrating the benefits of PA on pain, mood and flexibility over NSAIDs on the computer screen. While ExPa uses the patient’s registered pain intensity for visual presentation, it does not adjust its specific recommendations based directly on pain ratings. Instead, the focus is on tailoring PA options to the patient’s preferences and readiness for activity, rather than pain alone. Next, physicians can talk about diverse PA options (e.g. everyday exercise, digital offers and preventive sports) with their patient using a clickable catalogue tailored to patient preferences and personalised goals can be discussed. At the end of the consultation, patients receive a handout summing up the options with access to further information (e.g. links) and a follow-up appointment is arranged. Please see Fig. 1 for an impression of “ExPa” and supplement 1 for a detailed description of ExPa in the TIDieR format (template for intervention description and replication) [14].

### Intervention group

Physicians received written instructions on ExPa and were able to watch corresponding instructional videos (3 videos, in total 6.32 min). If they had any questions or uncertainties, they could contact the research team. The intervention is described above (the digital communication support programme ExPa) and in supplement 1.

### Control group

Physicians in the control group advised their CBP patients as usual (“usual care”). Typically, this involves providing general guidance on pain management strategies including medication, PA and potential referrals to physiotherapy. Patients in the control group could also receive ExPa advice from their physician after the study.

### Rationale for sample size in the feasibility trial

We planned to recruit six physicians, each responsible for recruiting five patients. Given the nature of this feasibility study, the sample size was not calculated to prove effectiveness of ExPa. Instead, it was pragmatically determined to allow sufficient data collection to assess feasibility aspects [18]. This sample size also enabled a more intensive application of qualitative methods.

### Measures and data management

**Primary outcome**:

- Data on the procedures of the study: duration of recruitment, dropout rate, completion time and data collection rates.

**Secondary outcomes** (before and three months after the ExPa consultation):

- PA: International Physical Activity Questionnaire (IPAQ) [19].
- Pain intensity: Von Korff pain intensity and disability score [20].
- Quality of life: 12-item Short Form Health Survey [21].
- Mood: Hospital Anxiety and Depression Scale (HADS) [22].
- Project-tailored questionnaires (Table 1, Supplement 2).
- Qualitative data (semi-structured interviews with the intervention group).
  - Feedback on study procedures (e.g. recruitment, data collection, consultation flow).
  - Views on ExPa (e.g. usability, impact on motivation, pain and PA).

**Table 1:** The topic is written in bold letters followed by a sample question. More details on questionnaires can be found in supplement 2

Main topics of the project-tailored questionnaires / interview guides	
Patients	**Physicians**
**Prior clinical history**	**Visualization of pain**
First of all, I would be interested to know how long you have had back pain?	Have you used the thermometer to visualize the effect of movement on pain?
**Visualization of pain using a thermometer**	**Function “other benefits of exercise”**
How did you like the thermometer?	Have you used the “other benefits of exercise” function exercise (improved mood and mobility)?
**Presentation Of The Other Benefits Of Exercise (Mobility, Mood)**	**Doctor and patient information**
Do you remember if your doctor has shown you other benefits of exercise (improved mood and mobility)?	Have you used the patient information in the catalog for PA options?
**Opinion on handout**	**Handout**
Do you remember what was on the handout?	Have you printed a hand-out for patients?
**Comprehensibility of the programme**	**The effect on motivation, the atmosphere of the conversation and manageability**
Did you find the presentation in the programme easy to understand?	Did you find the programme easy to use?
**Motivation/behaviour change**	**Ideas to improve the programme**
Do you have the impression that this consultation has brought about a change?	What is missing/should be improved in the programme?
**Ideas to improve the app**	Would you use the programme in the future?
What should be improved in the programme?	
Would you recommend this form of consultation to a friend?	
**Opinions on the course of the study:**	
**General opinion on taking part in the study**	
How did you feel about participating in the study overall?	
**Effort involved**	
How did you feel about the effort involved in participating in the study? `	
**Ideas to improve the course of the study**	
What should be changed about the study?	

Data on the procedures of the study (primary outcome) were tracked throughout the study. This information was gathered using spreadsheets in Microsoft Excel 2019 (Redmond, Washington, US) to log the dates of recruitment, consultation, follow-up and data submission.

We used several validated questionnaires to assess the outcomes: *PA*: The IPAQ measures the frequency and duration of PA across various domains to estimate a total PA level in metabolic equivalent minutes (MET-minutes) [19]. *Pain intensity*: The Von Korff Scale assesses chronic pain through two components: pain intensity and pain-related disability. Patients rate their average, worst, and current pain, as well as the impact on daily activities, using a 0–10 scale [20]. *Quality of life*: The SF-12 is derived from the longer SF-36 and provides two scores: The Physical Component Summary (PCS) and the Mental Component Summary (MCS) [21]. *Mood*: The HADS is used to detect levels of anxiety and depression, with separate subscales (HADS-A, HADS-D) [22]. Please also see Table 1 for information on the questionnaires.

We managed and analysed data in Microsoft Excel 2019 (Redmond, Washington, US), SPSS (IBM, Chicago, US) and R (Rstudio 2023, R Core Team).

### Data analysis

#### Quantitative data

Descriptive statistics were performed for primary and secondary outcome measures. Homogeneity of the sociodemographic characteristics between the groups was assessed using Wilcoxon Rank Sum test and chi² test as appropriate.

#### Qualitative data

We analysed the transcripts thematically in six steps based on the method of Braun and Clarke [23, 24, 25]. Reason for choosing thematic analysis was its flexibility and exploratory focus, allowing us to remain open to new insights which may arise during the feasibility trial. We used an inductive approach to be able to adapt emerging themes and to identify challenges throughout the study. During the first steps of analysis we developed a codebook to analyse the data (codebook type of thematic analysis). This approach facilitated collaboration among our research team while maintaining consistency. However, the codebook remained adaptable, allowing us to introduce new codes and refine existing ones as needed during the analysis.

We also considered disadvantages of thematic analysis, such as its potential subjectivity and the challenge to generalise findings.

Our analysis proceeded as follows: After the researchers had familiarized themselves with the transcripts, a preliminary coding (first version of the codebook) was developed based on three transcripts, which was checked within the research group using further transcripts. The content was fully coded in this way and preliminary themes were formed from it. Potential relationships between the themes (and possibly sub-themes) were noted. The research group iteratively developed themes, subthemes and relationships until an appropriate thematic description of the data was available. The data were analysed using the computer software MAXQDA version 2022 (VERBI Software, Berlin, Germany).

## Results

### Sample characteristics

Most of the included patients had CBP for more than 10 years and visited their doctor frequently because of back pain. Median pain intensity (Von Korff scale) in the intervention group was 60 [44.25–71.5] and in the control group 65 [52–75] (please see Table 4). Sociodemographic characteristics of patients showed no statistical difference between intervention and control group. An overview of baseline characteristics of included patients and physicians can be found in Table 2.

**Table 2:** Baseline characteristics of participating patients and physician

**a)**		
Baseline characteristics of participating patients		
	Intervention (*n* = 14)	Control (*n* = 17)
Age median [range]	59 [36–83]	57 [21–81]
Gender	Men: 9, Women: 5	Men: 4, Women: 13
Back pain duration	< 1 year: 0 1 year-10 years: 2 > 10 years: 12	< 1 year: 1 1 year-10 years: 8 > 10 years: 8
Number of doctor visits because of back pain in the past 12 months	< 4 times: 9 4–12 times: 5 > 12 times: 0	< 4 times: 3 4–12 times: 12 > 12 times: 2
**b)**		
Characteristics of participating physicians/surgeries		
	Intervention (*n* = 4)	Control (*n* = 4)
Age median [range]	39.5 [28–64]	53 [36–57]
Gender	Men: 3, Women:1	Men: 2, Women: 2
Duration of working in primary care in years [Interquartile range]	6.25 [1.375]	19 [11.25-25]
Type of surgery	Joint practice: 4	Joint practice: 4
size of surgery	Big: 4 (> 1500 patients per year)	Big 4 (> 1500 patients per year
Location of surgery	Urban: 1, rural: 3	Rural: 3, ng: 1

### Primary outcome: duration of recruitment and dropout rate

**Fig. 2:**
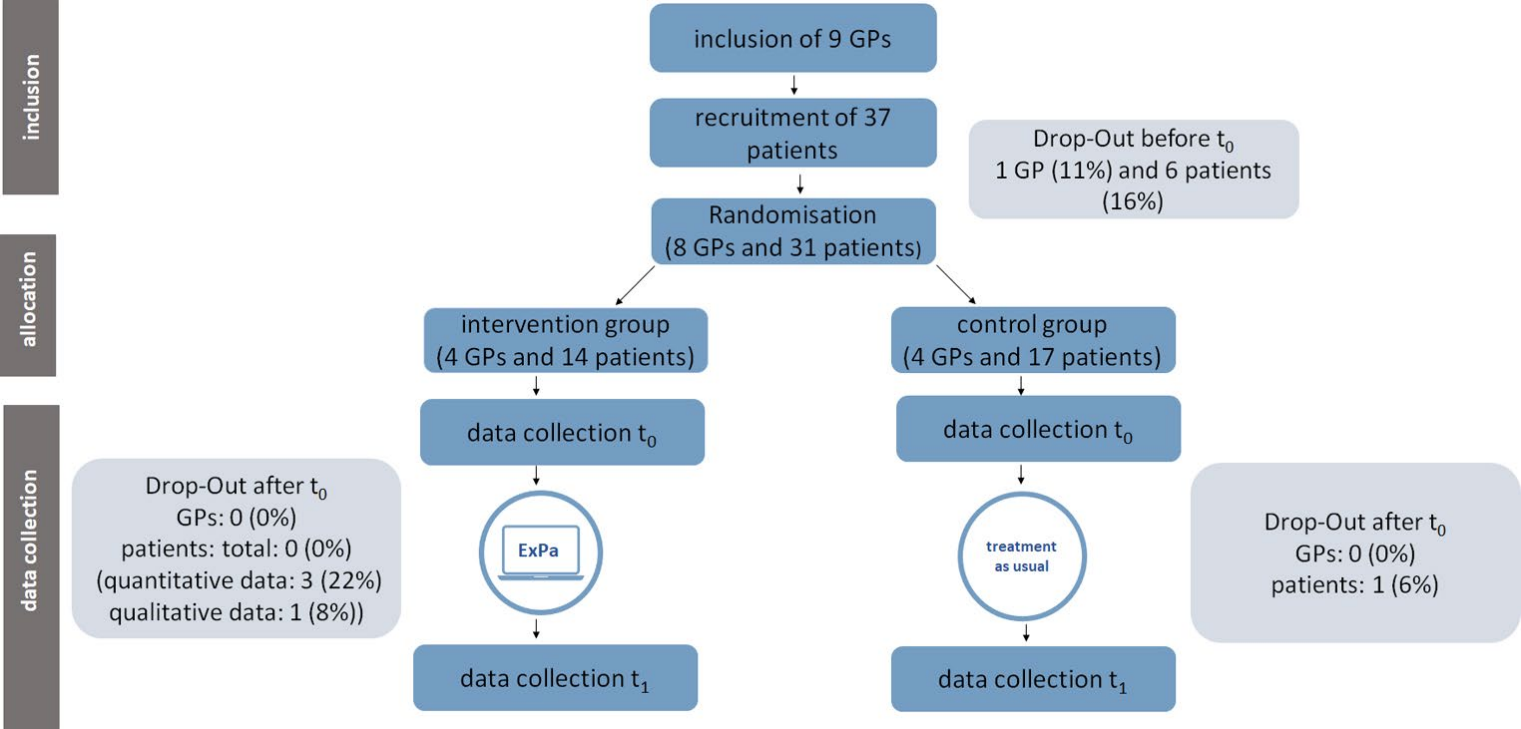
Participant flow diagram: t_0_: baseline survey, t_1_: survey after three months

We recruited participants from March 2022 to July 2022. Nine physicians (three women and six men) were recruited who then recruited 37 patients (18 women and 14 men). One physician (11%) and 6 patients (16%) could not be randomised because of a failure to contact them again. Physicians took an average of 38.5 days to recruit patients, exceeding the planned 21 days, which required extending the recruitment period. Please see Table 3 for detailed data on study procedures. Four physicians and their 14 recruited patients were randomly assigned to the intervention group, four physicians and 17 patients to the control group. All of the participants completed the baseline survey (t0) and all of the 14 patients in the intervention group underwent an ExPA guided consultation (in the median 35.5 days (range: 6 to 87 days) after consent to participate). Interviews were conducted with all phyisicans and 13 patients, mostly by telephoe (11 by phone, 1 in person). One patient refused to be interviewed because of pain exacerbation. At t1, a total of 27 patients had completed the survey, 3 patients of the intervention group (22%) and one patient of the control group (6%) could not be reached again. We collected data from all intervention group patients at t1, either through interview or questionnaires. All of the four physicians in the intervention group completed the survey, while data collection from the physicians in the control group was not necessary. Figure 2 provides an overview of the participant flow.

**Table 3:** Summary of study procedures

Overview of data on study procedures		
	Actual time	Planned time
Duration of recruitment of 6 physicians	50 days	63 days
Duration of recruitment of all 9 physicians	123 days	we planned to recruit only 6 physicians
Duration of recruitment of the first patient by physicians	median: 28.5 days, range: 3–51	na
Duration of recruitment of all patients by physicians	Median: 40.5 Range: 6–66	21 days for each physician
Number of patients recruited per physician	− 3 physicians recruited 4 patients each − 5 physicians recruited 5 patients each	5 patient per physician
Time until consultation took place	Median: 35.5 Range: 6–87	na

### Primary outcome: data collection rates

Missing data in the validated questionnaires (SF-12, IPAQ, HADS, von Korff) was considerable, particularly for PA (only 50% of the patients in the intervention group completed the IPAQ correctly) (Please see Table 4). However, interview feedback indicated that participants found the questionnaires manageable and not time-consuming. Only a few noted difficulties, such as assigning values to pain or expressed preference for electronic questionnaires.

**Table 4:** IPAQ is presented as total MET (metabolic equivalent) minutes. HADS: hospital anxiety and depression score, A: Anxiety-Subscale, D: Depression-Subscale, IPAQ: International Physical Activity Questionnaire, ng: not given, SF-12 MCS: short form 12 Health Survey, Mental Component score, SF-12 PCS: short form 12 Health Survey, physical component score

Outcomes assessed at T0 (baseline) and T1 (3 months after)			
	T0 Median Interquartile range in []	T1 Median Interquartile range in []	Missing cases
**Von Korff pain graduation** (0 (no pain)- 4 (high pain-related impairment)	Intervention:3 [3–4]	Intervention: 2 [1–3]	5
	Control: 3.5 [3–4]	Control: 2 [1–3]	1
**Von Korff pain intensity** (0 (no pain)– 100 (highest imaginable pain)	Intervention: 57 [43-65.5]	Intervention: 56.66 [41.5–64]	3
	Control: 66 [54.25–75.75]	Control: 59.8 [47.25–69.75]	1
**SF-12 PCS** (0 (lower)– 100 (higher physical related quality of life))	Intervention: 40 [37–43]	Intervention: 33.72 [33.23–40.24]	6
	Control: 30 [28–36]	Control: 33.6 [27.1–40.7]	5
**SF-12 MCS** (0 (lower)– 100 (higher mental health related quality of life))	Intervention: 39.5 [36-42.75]	Intervention: 48.81 [35.8-55.18]	6
	Control: 45 [34-55.5]	Control: 39.61 [33.92–54.31]	
**IPAQ** total MET (metabolic equivalent) minutes	Intervention: 7937 [2445–14239]	Intervention: 7572 [5412–10986]	9
	Control: 1413 (689–2278]	Control: 14,125 [953–1619]	7
**HADS-A** 0 (lower anxiety)- 21 (stronger anxiety)	Intervention: 5 [5-10.75]	Intervention: 8 [4.5–11]	4
	Control: 9 [6.75-11]	Control: 9 [5-10.25]	1
**HADS-D** 0 (lower depression)-21 (stronger depression)	Intervention: 7 [6–8]	Intervention: 6 [5-9.5]	3
	Control: 6 [4-8.25]	Control: 7 [4-8.25]	1

### Primary outcome: satisfaction with study procedures

Patients (median 7.59/ scale 0–10) and physicians (median 8/ scale 0–10) generally rated the study participation and effort positively. Qualitative interviews confirmed satisfaction with time and effort required. However, physicians noted that recruiting patients and organizing appointments was time consuming.

**Fig. 3:**
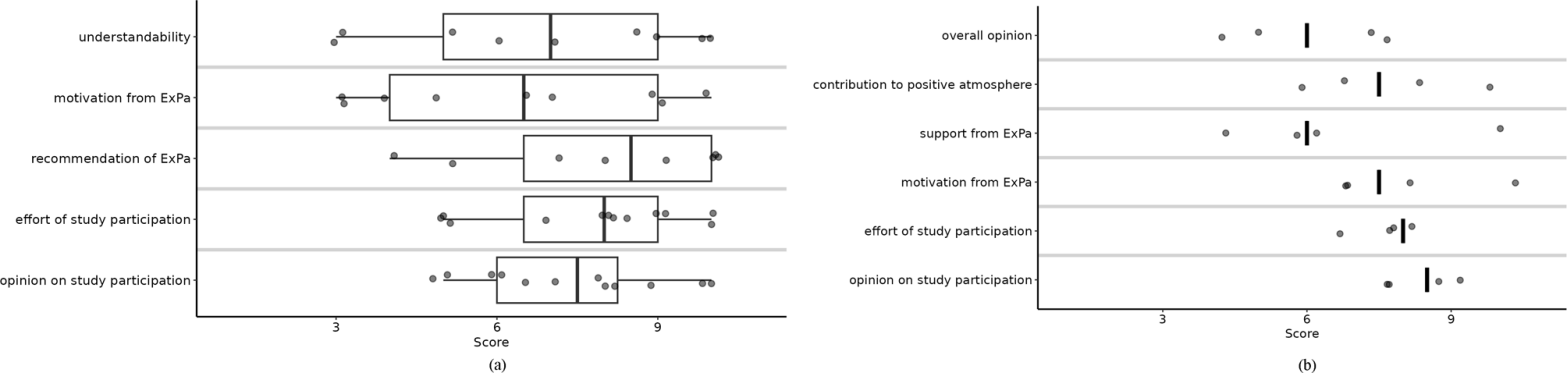
**a**: Boxplot on patients view on “ExPa”. Dots = individual data points, vertical line = median, box = interquartile range, horizontal line = whisker-line (1,5xinterquartile range). Figure 3**b**: Overview of physicians view on “ExPa” and study participation. Dots = individual data points, vertical line = median

Figure 3 provides an overview of views on ExPa and the study participation.

### Secondary outcome: PA and pain in the intervention group

Patients were satisfied with the counselling with ExPa (median 7/ scale 0–10), felt positive (median 7/ scale 0–10) and motivated (median 6.75/ scale 0–10). Patients partly expressed an improvement of pain (5 patients expressed a decrease of pain, 3 patients no change and 6 patients did not provide an answer). The majority would recommend the counselling with ExPa (median 8.5/ scale 0–10). This was also confirmed by the results of the qualitative interviews: Some patients expressed a greater sense of acceptance of their symptoms.

“*I felt more accepted*,* I must say*,* with my complaints. And I actually found that positive*” (patient_07).

The discussion atmosphere was seen as “*pleasant*” (patient_25) or at least as “*quite normal*” (patient_36) by patients as well as physicians. The main message of the counselling was conveyed to the patients:

“*So (…) drugs****don’t****have an effect as good as exercise*,* that is what I remember*” (patient_07).

Many patients reported at least small changes in their PA directly after the consultation. They for example, started to cover more distances on foot or signed in a course:

“*And then we came to the conclusion that I would prefer to do a course at home. And she [physician] prescribed it for me. And I had it approved by my health insurance*” (patient_07).

Please see Fig. 3 for overview of results regarding the view of patients on ExPa.

### Secondary outcome: ExPa contributed to a positive motivating consultation

Physicians generally felt somewhat supported by ExPa (median 6/ scale 0–10) and believed it contributed to a positive patient-physician relationship (median 7.5/ scale 0–10). They found the consultation motivating for for patients (median 7.59/ scale 0–10). In the interviews, physicians stated that they found ExPa helpful to structure their counselling:

“*So I found that quite good as a guideline*” (physcian_01).

They took more time to talk about PA with their patients and learned new aspects about them. Please see Fig. 3 for an overview of physicians view on ExPa.

### Secondary outcome: mixed feedback and suggestions for optimizing ExPa

Satisfaction with using ExPa again was mixed (median 5 on a scale 0–10) and some features were rarely used. Also, in the interviews, few patients and physicians said that they did not to see additional benefits from using ExPa. They found the conversation partly too short:

“*So it was surprisingly short. And for me it was also rather disappointing*,* I must say. I actually already knew what I was told*” (patient_03).

In some cases, the graphical representation was found to be unconvincing. Some patients noticed the physicians´ uncertainties on using ExPa correctly:

“*So I think Dr (…) is still having a bit of trouble with the programme*.” (patient_23).

Sometimes physicians did not show them the monitor or they did not use the function to print handouts. Physicians criticized ExPa for being time consuming and hard to integrate it in the practice routine. As a solution, they suggested the implementation of ExPa in the practice software.

On the other hand GPs as well as patients expressed also additional topics, which should be integrated into ExPa:

“So *let’s say the occupational area or psychosocial stress factors… these should at least appear in a list like this*,* so that they are discussed* (physician_09).

### Overview of results of the qualitative analysis

Interviews with the patients and physicians of the intervention group revealed the following themes: [1] view on study procedures [2], insights into the consultation process [3], views on ExPa [4], consultation atmosphere [5], challenges and disadvantages of using and implementing ExPa [6], ideas for improvement [7], impression of specific features of ExPa and [8] changes after the consultation with ExPa. Since this study employed a mixed-methods approach, we have presented the qualitative and quantitative results in the relevant sections above. For an overview of study results sorted by theme please see supplement 3.

## Discussion

### Summary

The aim of this study was to evaluate the feasibility of a study using the consultation app ExPa to support PA consultations for CBP. Regarding the primary outcome the study procedures were generally feasible as planned. However, several important findings emerged for planning a larger RCT. Specifically, there was a high rate of missing data and extended study procedures. The study procedures have to be refined for a future bigger RCT. In terms of secondary outcomes, the results were promising. ExPa could potentially decrease pain and increase PA, with positive feedback form participants. However, these findings need to be confirmed by a larger RCT.

### Comparison with existing literature

The positive effects of ExPa on PA and pain align with previous research demonstrating the potential of digital interventions to improve these outcomes. Several systematic reviews and meta-analysis have highlighted the beneficial impact of digital interventions on pain [26, 12]. However, they also emphasise the need for further research on digital interventions that can be effectively implemented in clinical workflows [12].

PA consultation with patients with CBP can sometimes lead to tension in the doctor-patient-relationship and frustration on both sides [11]. Therefore, the contribution of ExPa to a positive and motivating atmosphere and the insight that patients felt more accepted is of great importance. In this context, ExPa could have great potential.

Although our feasibility study demonstrated satisfaction with ExPa and study procedures among physicians and patients, the dropout rates for quantitative and qualitative data combined in the intervention group was considerable. Because of cluster randomisation some patients needed to wait several weeks before the consultation with ExPa, which may have contributed to the high dropout rate in the intervention group. However, compared to other digital interventions such as app-based interventions for different chronic diseases with a pooled dropout of 43%, the observed dropout in our feasibility study seems to within in normal range [27]. Yet, the validity of a bigger RCT could be affected in particular when the dropout-rates differ between the groups [28].

In addition, the rate of missing data was considerable. The inclusion of numerous questionnaires may have contributed to participant burden and potentially affected the completion rates. In the context of IPAQ, O’Neill et al. described that in their study of people with bronchiectasis most of the patients required assistance to complete the IPAQ questionnaire [29]. This finding is in line with qualitative results, which showed that there are problems in understanding and correctly completing the questions of the IPAQ, especially in the older population [30]. To address this, telephone data collection could be a reliable alternative to face-to-face methods [31]. Furthermore wearable devices, such as ActiGraph, may provide a more objective measure [32]. However, the data on validity varies depending on factors like the specific device, analysis algorithm, walking speeds and position of device [33]. Moreover, the data on user acceptability ranges from positive (e.g. motivation) to negative experiences (e.g. anxiety) [34].

### Strengths and limitations

A strength of this feasibility study is the gain of valuable insight into the study flow und procedures. We could identify areas, which need improvement in the development a full effectiveness trial. Furthermore, the consultation software ExPa was acceptable for physicians and patients and might improve patient-physician relationships and lead to increased PA as well as decreased pain.

Some limitations have to be considered:

Due to a lack of resources and the type of intervention, blinding was not possible. Patients will likely guess to which group they were allocated to and physicians will always be unblinded from the start. However, in the full RCT, we plan to blind research assistant collecting data as well as include a blinded statistician in the research group.

A key limitation is the high rate of missing data, which hinders our ability to draw conclusions about the effects on outcomes like PA. This limits the interpretability of the quantitative findings.

Another limitation is that characteristics on baseline PA, mental and physical capacity between the intervention and control group were not well balanced. Specifically, patients in the intervention group appeared to have higher baseline PA levels (as measured by the IPAQ) and lower physical quality of life scores (as measured by the SF-12 PCS). This imbalance could have influenced the outcomes, as those in the intervention group may have already had a positive experience with PA. However, due to the missing data, it is challenging to draw definitive conclusions. A future RCT should consider stratifying groups.

An additional limitation of this study is the inclusion of only physicians, excluding other healthcare professionals like physiotherapists or nurses. This restricts insights into how ExPa could be applied across diverse clinical roles and settings.

It is possible that participants changed their behaviour because they were in a study (Hawthorne effect) [35]. Included physicians in the control group might provide more advice on PA and patients in the control group might do more PA, knowing that the study was focused on PA and CBP.

### Implications for research and practice

The findings of this feasibility study will inform the design of an upcoming effectiveness trial. This larger trial will aim to evaluate the impact of ExPa intervention on PA and in patients with CBP. Key adjustments to the trial design will include overworking data collection methods to minimise missing data, speeding up the trial duration for the individual patient to reduce drop-out, and revising ExPa based on the feedback received.

The rate of missing data by just sending the questionnaires to participants was high, data collection should be completed either in person or by telephone. Alternatively, an electronic case form could be used, requiring participants to complete all questions before being able to proceed and finalize the questionnaire. Fitness trackers could be used to collect PA data, though devices have different advantages and disadvantages. A careful decision has to be made if trackers are suitable for the study.

The total dropout rate was higher in the intervention group than in the control group. In an RCT recruitment of participants would need to be faster, as some had to wait several weeks before the ExPa consultation took place. They might have improved their back pain or lost their interest in the study during that time. A possibility would be to reduce the number of patients an individual physician has to recruit, so that the ExPa consultation can take place earlier.

Based on the qualitative and quantitative feedback received, we refined the ExPa programme, e.g. incorporating information on psychological and occupational issues, revising the training for physicians and facilitating the access to ExPa.

Some physicians had problems to use ExPa. To avoid technical uncertainties, the programme should be presented to the physician in person with supporting material such as videos and instructions that can be used any time. It would make sense to offer standardized training for all participating physicians. In the context of a larger RCT, an accompanying process evaluation should be carried out to find out whether the intervention was adhered to and which mechanisms and contexts are necessary to implement the intervention successfully. In addition, a health economic analysis is necessary to find out whether the implementation of ExPa can reduce direct and indirect health care costs. For this analysis, it would be beneficial to collect more detailed data on the control group, specifically regarding the type of usual care they received. Similarly, for the intervention group, it would be important to track whether patients sought additional healthcare services.

As an implication for clinical practice the results of this study (in particular the qualitative interviews and the project-tailored questionnaires) show that the digital consultation programme can potentially be effective. To investigate the efficacy of ExPa, the study design must first be adapted according to the results of our feasibility study in order to then plan a larger randomized controlled effectiveness trial. Regarding ExPa’s clinical implication, it could also be utilised by other healthcare professionals, such as physiotherapists or pain management specialists, thereby extending its reach to support patients across various health care settings.

## Conclusions

Our study provides important information for conducting an RCT; recruitment periods must be adjusted and it must be determined how relevant outcomes (such as PA and pain) can be collected effectively with limited loss of data. We cannot draw definitive conclusions regarding our secondary outcomes. However, findings in this study shows indications of that the implementation of ExPa reduces pain and increases PA in the intervention group.

## Electronic supplementary material

Below is the link to the electronic supplementary material.

Supplementary Material 1

## Data Availability

The datasets used and/or analysed during the current study are available from the corresponding author on reasonable request.

## Abbreviations

CBP: Chronic back pain
ExPa: Exercise against Pain
MRC: Medical Research Council
PA: Physical activity
RCT: Randomised controlled trial
US: United States of America

## Declarations

### Presentations prior publication

Preliminary results were presented at the DEGAM conference 2022.
