# Supplementary material for: Digitally supported physical activity counselling for people with chronic back pain: a randomised controlled parallel feasibility study
Source: BMC Prim Care. 2025 Feb 27;26:58. doi: 10.1186/s12875-025-02742-z (PMC11866899; doi:10.1186/s12875-025-02742-z)
Supplement: Supplementary file 1 — Supplementary Material 1 [file 12875_2025_2742_MOESM1_ESM.docx]

**Supplement 1: Description of ExPa with TIDieR**^1^

**BRIEF NAME**

1. *Provide the name or a phrase that describes the intervention.*

ExPa (Exercise and Pain)

**WHY**

1. *Describe any rationale, theory, or goal of the elements essential to the intervention.*

Previous approaches to the therapy of chronic back pain have been ineffective. There is no successful initiation of physical activity therapy for chronic back pain in primary care practices. ExPa aims to fill this gap.

**WHAT**

1. *Materials: Describe any physical or informational materials used in the intervention, including those provided to participants or used in intervention delivery or in training of intervention providers. Provide information on where the materials can be accessed (e.g. online appendix, URL).*

Consultation using the digital physical activity counseling program ExPa by the general practitioner.

Duration of consultation: 15 minutes

Material: online accessible counseling program ExPa with

- Training material for the primary care doctor to improve communication and shared decision-making (SDM)
- Counseling program for advising patients

1. *Procedures: Describe each of the procedures, activities, and/or processes used in the intervention, including any enabling or support activities.*

- Primary care physicians use training material to improve communication/SDM
- Primary care physicians advise their patients with chronic back pain during the consultation using ExPa:

1.⁠ ⁠Presentation of the positive effect of physical activity on pain using a thermometer.

2.⁠ ⁠Comparison with the lower effect of NSAIDs.

3.⁠ ⁠Presentation of the positive effect of physical activity on mood and mobility

4.⁠ ⁠Discussion of physical activity options using a catalog with several clickable fields

e.g.: everyday movement, digital offers, rehabilitation sports, prevention sports, back exercises.

Information for physicians and patients is provided here.

5.⁠ ⁠Setting joint goals.

6.⁠ ⁠Hand-out for the patient with individualized information.

7.⁠ ⁠Follow-up appointment after three months.

**WHO PROVIDED**

1. *For each category of intervention provider (e.g. psychologist, nursing assistant), describe their expertise, background and any specific training given.*

Primary care doctors with different backgrounds (duration of general practice activity, setting (urban/rural, group practice/solo practice))

**HOW**

1. *Describe the modes of delivery (e.g. face-to-face or by some other mechanism, such as internet or telephone) of the intervention and whether it was provided individually or in a group.*

The counseling using ExPa takes place as part of an appointment planned for this counseling with the primary care doctor during regular office hours.

**WHERE**

1. *Describe the type(s) of location(s) where the intervention occurred, including any necessary infrastructure or relevant features.*

The counseling takes place in the regular practice in the doctor's office using a computer.

**WHEN and HOW MUCH**

1. *Describe the number of times the intervention was delivered and over what period of time including the number of sessions, their schedule, and their duration, intensity or dose.*

The counseling using ExPa is primarily done once. Subsequently, a follow-up appointment is scheduled to reevaluate the individually discussed movement options and goals. The ExPa program can be used again during the follow-up appointment.

**TAILORING**

1. *If the intervention was planned to be personalised, titrated or adapted, then describe what, why, when, and how.*

The counseling is individually tailored to each patient:

- Adjustment to the individual pain level of the patient
- Individualized movement options can be discussed and presented
- Individual goals are set

**MODIFICATIONS**

1. *If the intervention was modified during the course of the study, describe the changes (what, why, when, and how).*

Based on the results of the feasibility study and feedback from the patient council, the presentation in the thermometer has been adjusted, and options for movement have been expanded to include information on the workplace and mood. During the feasibility study the ExPa intervention was not changed.

**HOW WELL**

1. *Planned: If intervention adherence or fidelity was assessed, describe how and by whom, and if any strategies were used to maintain or improve fidelity, describe them.*

Change in physical activity, measured using IPAQ (International Physical Activity Score), project-tailored questionnaires, qualitative interviews

1. *Actual: If intervention adherence or fidelity was assessed, describe the extent to which the intervention was delivered as planned.*

Due to missing data the IPAQ could not not be analysed robustly, according to qualitative data und project-tailored questionnaires physical activity may have increased

1. Hoffmann TC, Glasziou PP, Boutron I, et al. Better reporting of interventions: template for intervention description and replication (TIDieR) checklist and guide. *BMJ*. 2014;348:g1687. doi:10.1136/bmj.g1687.

**Supplement 2:**

| **Project-tailored questionnaires (patients)** |
| --- |
| 1. Do you remember if your doctor showed you a thermometer to demonstrate the effect of exercise on pain? 2. If so, how did you like the thermometer? 3. Do you remember if your doctor has shown you other benefits of exercise (improved mood and mobility)? 4. If yes: How did you like the presentation with the traffic light man and smiley? 5. Do you remember if your doctor gave you a printout? 6. If yes: How did you like the printout? 7. Do you remember what was on the printout? (Selection of different options….) 8. Have you used a link of the printout? 9. Did you find the presentation in the arriba tool easy to understand? 10. Did you find the consultation with the arriba tool motivating? 11. How would you rate the advice provided by the arriba programme overall? 12. Do you have the impression that this consultation has brought about a change? 13. Would you recommend consulting with the arriba program? 14. What is missing/should be improved in the tool? 15. How did you feel about participating in the study overall? 16. How did you feel about the effort involved in taking part in the study? 17. What should be changed about the study? |

Project tailored questionnaires patients. 1., 3., 5., 8.: two answer options (yes or no),7., 12.: Selection of different options, 14., 17.: free text, The remaining questions were answered using a Likert scale

| **Project-tailored questionnaires (doctors):** |
| --- |
| 1. Have you used the thermometer to visualize the effect of movement on pain? 2. How did you like the thermometer? 3. In your opinion, did the visualization of the effect of movement on pain support the consultation? 4. Have you used the "other benefits of exercise" function? 5. How did you like the "other benefits of exercise" function? 6. Have you used the doctor information in the catalog for different options? 7. How did you like the doctor information? 8. Have you used the patient information in the catalog for various options? 9. How did you like the patient information? 10. Have you printed a hand-out for patients? 11. How did you like the hand-out? 12. Have you taken the opportunity to add your own free text under "local offers"? 13. Did the arriba tool help you with your movement consultation? 14. Did the arriba tool contribute to a positive discussion atmosphere? 15. Did you have the impression that the patient was motivated by this form of counseling? 16. Did you find the arriba programme easy to use? 17. What is your overall assessment of the arriba exercise counseling program? 18. Would you use the arriba tool for movement counseling again? 19. What is missing/should be improved in the tool? 20. We are also interested in your opinion on the conduct of the study. How did you feel about the effort involved in participating in the study? 21. How did you feel about participating in the study overall? 22. What should be changed in the study? |

Project tailored-questionnaires doctors: 19., 22.: Free-text, 12.: two answer options (yes or no), the remaining questions were answered using a Likert Scale.

**Supplement 3**

| Overview of results of the qualitative analysis |
| --- |
| (1) view on study procedures  patient_24: *"It was actually manageable. I wouldn’t say that it was very, very time-consuming."*  patient_36: *"Just digital [the questionnaires]. It’s easier."*  physician_02: *"Yes, overall, I think it’s well organized."* |
| (2) insights into the consultation process  physician_02: *"I really liked that at the beginning, the pain was assessed using the numerical analogue scale. Yes, visualizing the pain level was helpful. It got really interesting when we moved on to: 'Okay, what can be done about it?’”*  physician_01: *"I thought it was quite good as a guideline, ensuring that nothing gets overlooked."*  patient_07: *"Well, we discussed what would be most suitable for me, what she or I thought would be best. And she went through a checklist for that.“*  patient_28: *"We talked about it in general terms, but I can’t really remember the details anymore."* |
| (3) views on ExPa  patient_03: *“I don’t know what it does for others, but for me, it was fine.”*  patient_21: *“I would say, these (…) system, I thought—, I think it is great.”* |
| (4) consultation atmosphere  patient_25: *"Yes, it was a pleasant conversation."*  patient_24: “*I would say it was actually a very relaxed patient-doctor conversation.”* |
| (5) challenges and disadvantages of using and implementing ExPa  patient_23: *“So I think Dr (…) is still having a bit of trouble with the programme."*  patient_22: *“And then the conversation was over in no time.”*  patient_38: *"What I was told, I actually already knew."*  physician_09: *"Time is a very scarce resource for us. That’s why we usually don’t have as much time to chat with patients as we did during this study."* |
| (6) ideas for improvement  patient_25: *"I think it’s definitely important to always consider the psychological background as well."*  physician_09: *"Well, I’m not sure, but maybe the work-related aspect isn’t covered here. Let’s say, the occupational area or psychosocial stress factors, which can have a negative impact, should definitely be included."* |
| (7) impression of specific features of ExPa  patient_03: *"Well, for me, it [sheet with back exercises] was beneficial. I’ve been doing it twice a day for at least three weeks now, and it’s really made a difference for me."*  patient_07: "*I thought it [visual presentation of pain] was basically quite good. I wouldn’t know how else it could be presented."* |
| (8) changes after the consultation with ExPa  patient_07: *"And then we came to the conclusion that I would prefer to do a course at home. She wrote that down for me, and I got it approved by my health insurance.”*  *patient_23: "Sometimes you get a little nudge towards something you might not have thought of, and that’s definitely not a bad thing."*  patient_28: *"I will definitely do that [PA]; it’s a great suggestion.”*  physician_01: *"For example, it was the first time I prescribed this digital health application (DiGA) for back pain."*  physician_01: *“I did have the impression that most of them left feeling quite motivated."*  physician_11*: “I had patients who were already motivated, and they embraced it very well. But then there were others who, and this might be a prejudice, but if you already think, okay, they’re probably not that active to begin with—those who may have done certain things in the past but no longer do so—those patients didn’t show much interest in doing more."* |
